# Normal tension glaucoma-like degeneration of the visual system in aged marmosets

**DOI:** 10.1038/s41598-019-51281-y

**Published:** 2019-10-16

**Authors:** Takahiko Noro, Kazuhiko Namekata, Atsuko Kimura, Yuriko Azuchi, Nanako Hashimoto, Keiko Moriya-Ito, Yuji Komaki, Chia-Ying Lee, Norio Okahara, Xiaoli Guo, Chikako Harada, Euido Kim, Tadashi Nakano, Hiroshi Tsuneoka, Takashi Inoue, Erika Sasaki, Hironobu Tokuno, Takayuki Harada

**Affiliations:** 1 Visual Research Project, Tokyo Metropolitan Institute of Medical Science, Tokyo, Japan; 2 Central Institute for Experimental Animals, Kawasaki, Japan; 3 Department of Ophthalmology, The Jikei University School of Medicine, Tokyo, Japan; 4 Center for Basic Technology Research, Tokyo Metropolitan Institute of Medical Science, Tokyo, Japan; 5 Laboratory of Brain Structure, Tokyo Metropolitan Institute of Medical Science, Tokyo, Japan

**Keywords:** Retinal diseases, Experimental models of disease

## Abstract

The common marmoset (*Callithrix jacchus*) is a non-human primate that provides valuable models for neuroscience and aging research due to its anatomical similarities to humans and relatively short lifespan. This study was carried out to examine whether aged marmosets develop glaucoma, as seen in humans. We found that 11% of the aged marmosets presented with glaucoma-like characteristics; this incident rate is very similar to that in humans. Magnetic resonance imaging showed a significant volume loss in the visual cortex, and histological analyses confirmed the degeneration of the lateral geniculate nuclei and visual cortex in the affected marmosets. These marmosets did not have elevated intraocular pressure, but showed an increased oxidative stress level, low cerebrospinal fluid (CSF) pressure, and low brain-derived neurotrophic factor (BDNF) and TrkB expression in the retina, optic nerve head and CSF. Our findings suggest that marmosets have potential to provide useful information for the research of eye and the visual system.

## Introduction

The elderly population is growing worldwide as life expectancy drastically increases^1^, creating new challenges related to the fact that aging is a risk factor for a variety of chronic conditions, including neurodegenerative diseases^2^. The incidence rate of glaucoma, a neurodegenerative disease of the eye characterized by a slow progressive degeneration of retinal ganglion cells (RGCs) and their axons, also increases with aging^3^. Glaucoma is one of the major causes of blindness and it is estimated that this disease will affect more than 80 million people worldwide by 2020^4^. Unfortunately, current therapy is insufficient to restore sight lost due to glaucoma. The disease is usually associated with elevated intraocular pressure (IOP). The common adult-onset glaucoma is primary open-angle glaucoma (POAG), which is frequently caused by a reduction in outflow of aqueous humor through the trabecular outflow pathways^5^. Meanwhile, normal tension glaucoma (NTG), a subset of POAG that indicates statistically normal IOP, also shows glaucomatous optic neuropathy and a characteristic visual field defect. Several population studies have suggested that NTG represents 20%–90% of all POAG, with percentages seeming to vary according to race^6–8^. Since some glaucoma patients do not benefit from lowering of IOP, including NTG patients, factors other than elevated IOP may contribute to disease progress. Therefore, elucidation of such non-IOP dependent factors would be necessary to understand the pathogenesis of glaucoma and guide toward improved therapeutics.

In many studies, rodents have been the experimental animals of choice. Mouse models of disease are very useful and provide important information, but anatomical differences between rodents and humans mean that using a model more closely related to humans is desirable. With regard to glaucoma, we previously reported genetically modified mouse models of NTG^9,10^. These and other rodent models have contributed greatly to further understanding of this disease and exploring new therapeutic strategies^11^. However, one of the key structures involved in glaucoma, namely the lamina cribrosa (LC), is absent in mice, suggesting that use of animals that possess LC, such as non-human primates, could provide great value in glaucoma research.

The common marmoset (*Callithrix jacchus*), a small new world primate, is becoming increasingly attractive as an experimental animal model, particularly in neuroscience research^12^. Like humans, the common marmoset is diurnal, and its brain and eyes are structurally well developed. Their compact lifespan (10–15 years, with a maximum lifespan of approximately 20 years) allows monitoring of the effects of aging or progressive disease over a relatively short period of time, from 8 years of age onward^13^, making them ideal for aging research.

In this study, we examined the eyes of 36 aged marmosets and found that 11% of them presented with spontaneous NTG. This is the first study to thoroughly examine the glaucoma-like pathology in living marmosets and we found a high resemblance between marmoset and human glaucoma.

## Results

### NTG-like optic nerve and retinal degeneration in aged marmosets

**Figure 1:**
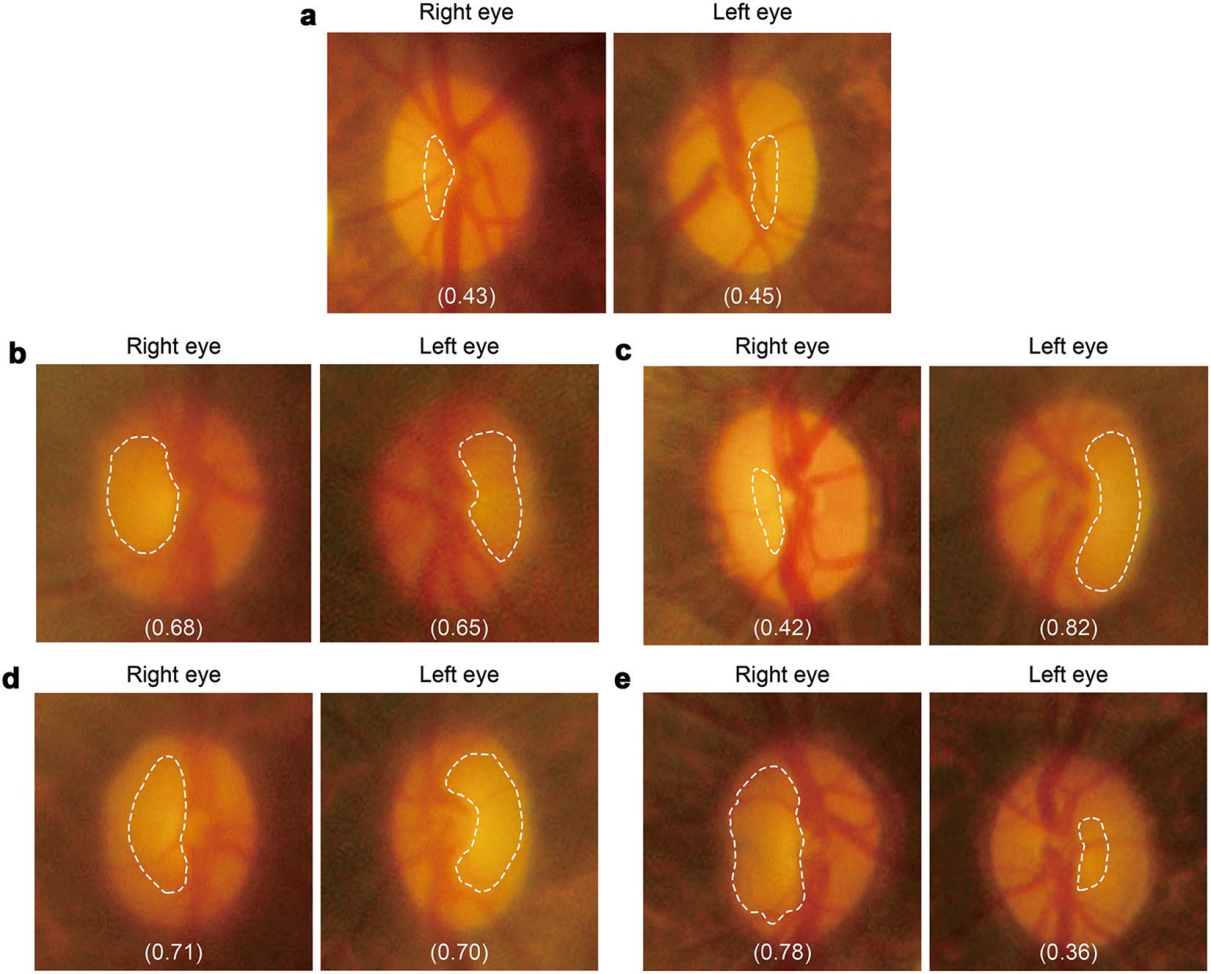
Evaluation of ocular fundus images of marmosets. **(a)** Ocular fundus photographs of aged control marmoset (No. 30 in Supplementary Table 1). **(b–e)** Ocular fundus photographs of glaucomatous marmosets. No. 33 **(b)**, No. 34 **(c)**, No. 35 **(d)**, and No. 36 **(e)**. Optic disc cuppings are outlined by dashed lines and the cup/disc ratio are shown in parentheses. The edge of the cupping was traced from the 3D images of the optic nerve head obtained by OCT and the lines were superimposed on the fundus photograph.

**Figure 2:**
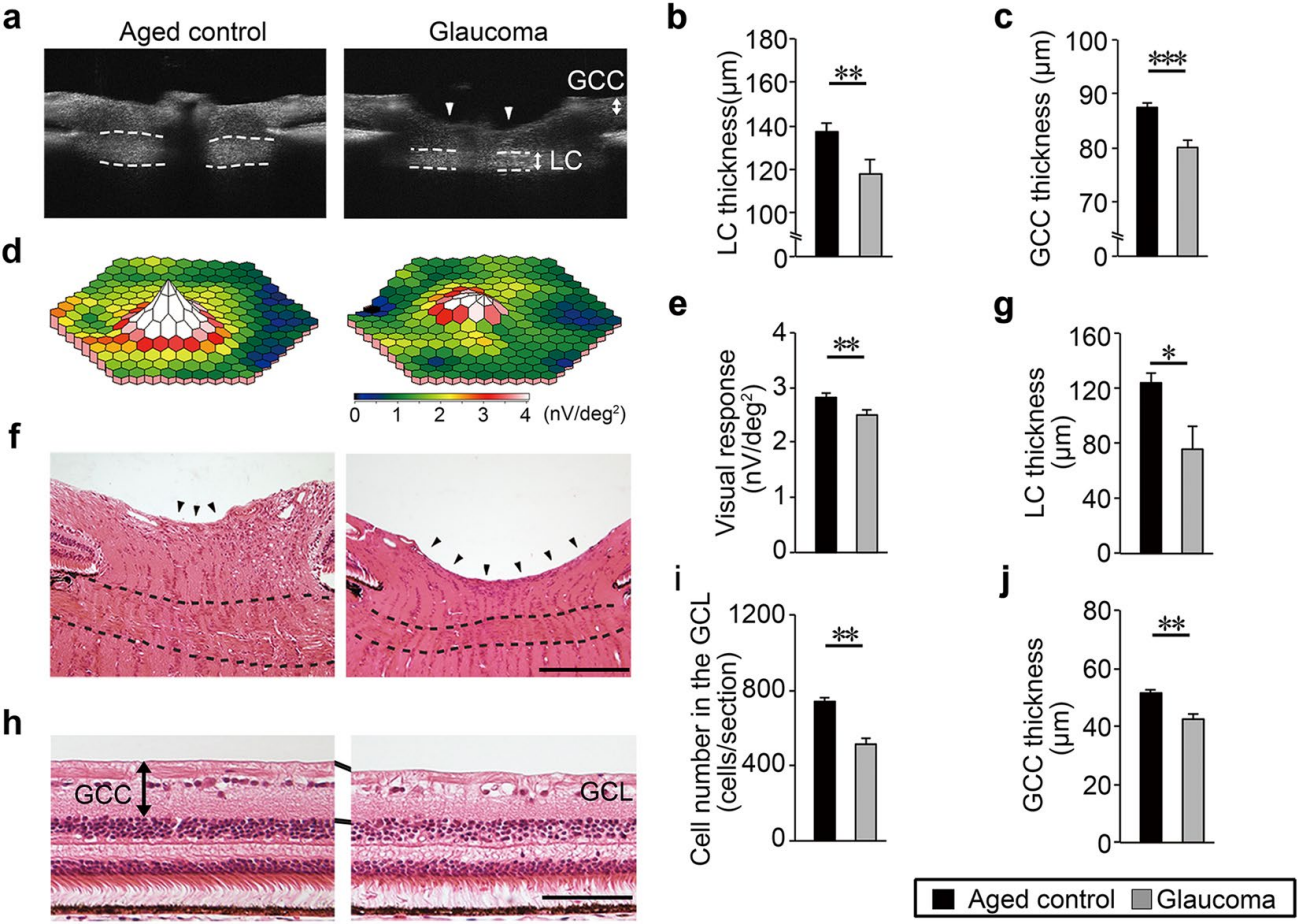
Degeneration of the optic nerve and retina, and impaired retinal function in glaucomatous marmosets. **(a)** *In vivo* imaging of the optic disc by vertical scan through the centre of the optic disc by SD-OCT. Arrowheads indicate the cupping of the optic disc and dotted lines indicate the LC. LC: lamina cribrosa, GCC: ganglion cell complex. **(b,c)** Quantitative analysis of the thickness of the LC **(b)** and GCC **(c)**. *n* = 28 (aged) and 6 (glaucoma). **(d)** Three-dimensional plots of the retinal responses as examined by multifocal electroretinogram (mfERG). A higher score (white) indicates highly sensitive visual function. Values are given in nV per square degree (nV/deg^2^). **(e)** Quantitative analysis of the visual responses. *n* = 28 (aged) and 6 (glaucoma). **(f)** H&E staining of the optic nerve head. Enhanced optic disc cupping (arrowheads) and thinning of the LC (dotted lines) are apparent in the glaucomatous marmoset. Scale bar: 200 µm. **(g)** Quantitative analysis of the LC thickness. *n* = 3 per group. **(h)** H&E staining of the retina. Inner retinal degeneration is observed in the glaucomatous marmoset. The mid-peripheral region is shown. Scale bar: 100 µm. GCL: ganglion cell layer. **(i,j)** Quantitative analysis of the cell number in the GCL **(i)** and GCC thickness **(j)**. *n* = 3 per group. The data are presented as means ± S.E.M. *P < 0.05; **P < 0.01, ***P < 0.001.

In this study, we monitored 36 aged (average age 11.3 years; Supplementary Table 1). Ophthalmoscopy was used for screening for increased excavation and thinning of the neuroretinal rim, which are hallmarks of glaucoma (dotted lines in Fig. 1a–e). The optic cup/disc ratio of >0.5 was subjected to further analysis by *in vivo* imaging with spectral-domain optical coherence tomography (SD-OCT). Strikingly, we found that out of 36 aged marmosets, 12 eyes (7 animals) presented with glaucoma-like characteristics, in which *in vivo* imaging with SD-OCT demonstrated that 6 eyes (4 animals) showed the cup/disc ratio of >0.65 (Supplementary Movies 1 and 2)^14^. These 4 marmosets are hereafter referred to as glaucomatous marmosets (Fig. 1b–e). SD-OCT demonstrated that the thinning of the LC and ganglion cell complex (GCC) were apparent in glaucomatous marmosets (Fig. 2a–c). In order to determine if the retinal degeneration reflects functional aspects, we investigated retinal function using multifocal electroretinogram (mfERG). The second-order kernel, which is impaired in patients with glaucoma, was analyzed as previously reported^9,15–17^. The response topography demonstrated that the average retinal responses of glaucomatous marmosets were reduced compared with controls (Fig. 2d,e). Histological examinations confirmed the thinning of the LC and GCC (Fig. 2f–j), which were consistent with the data from SD-OCT (Fig. 2a–c).

**Figure 3:**
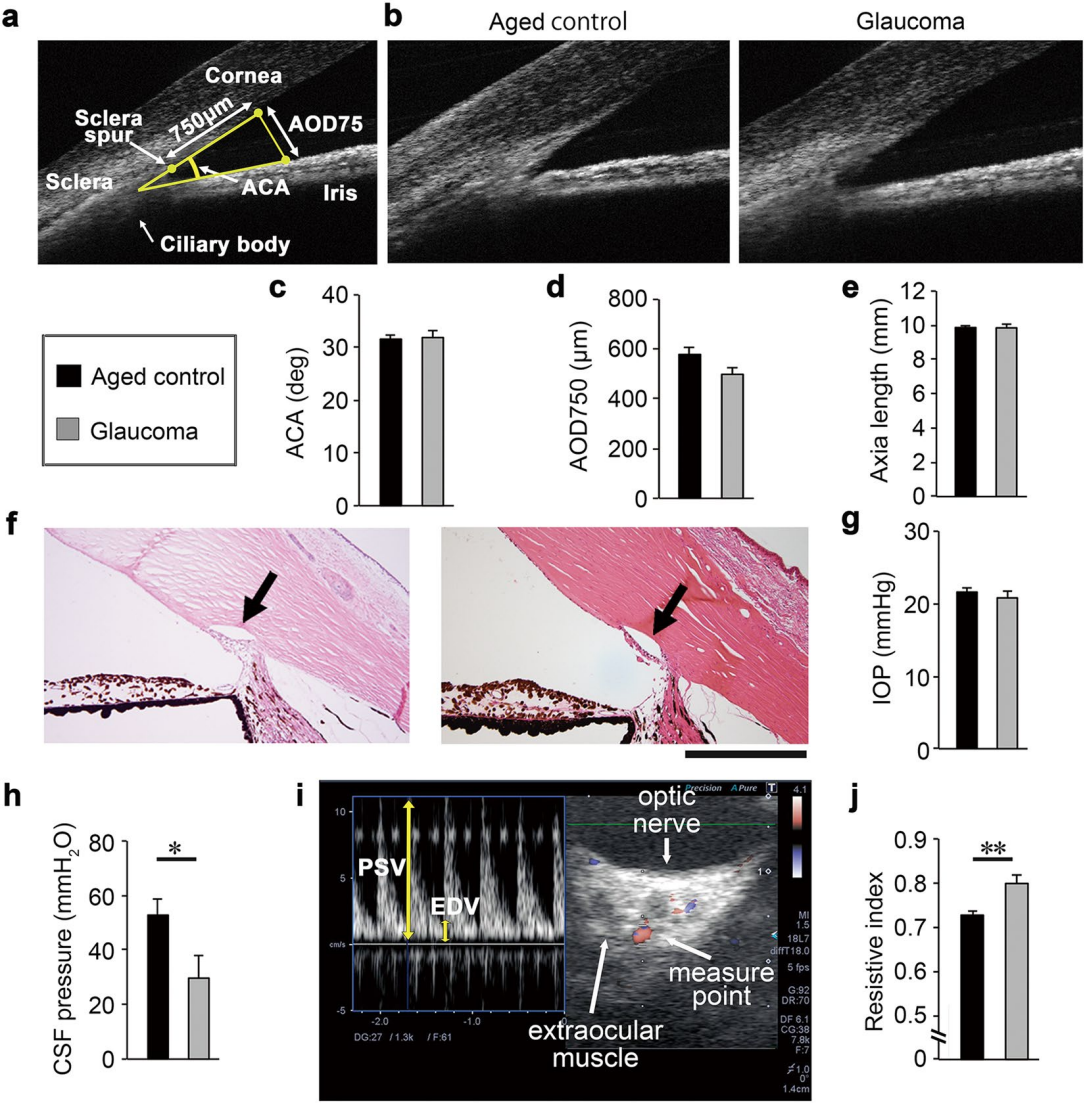
Pathological features resembling human normal tension glaucoma in aged marmosets. **(a,b)** *In vivo* imaging of the anterior chamber by SD-OCT. Illustration of the parameters measured in the anterior chamber **(a)**. The iridocorneal angle in the glaucomatous marmoset is wide open and comparable to that in aged marmoset **(b)**. ACA: anterior chamber angle, AOD: angle opening distance. **(c,d)** Quantitative analyses of ACA **(c)** and AOD750 **(d)** in aged and glaucomatous marmosets. *n* = 28 (aged) and 6 (glaucoma). **(e)** Quantitative analysis of the axial length in aged and glaucomatous marmosets using a color Doppler imaging scanner. *n* = 30 (aged) and 6 (glaucoma). **(f)** H&E staining of aqueous humor drainage structures in aged (left) and glaucomatous (right) marmosets. The iridocorneal angle in the glaucomatous marmoset is normal with an obvious Schlemm’s canal (arrow) and trabecular meshwork. Scale bar: 200 µm. **(g)** The IOP of aged and glaucomatous marmosets. IOP: intraocular pressure. *n* = 63 (aged) and 6 (glaucoma). **(h)** The CSF pressure of aged and glaucomatous marmosets. CSF: cerebrospinal fluid. *n* = 6 (aged) and 3 (glaucoma). **(i)** Representative images of the flow velocity of the ophthalmic artery using a color Doppler imaging scanner. PSV: peak systolic velocity, EDV: end diastolic velocity. **(j)** Quantitative analysis of the resistive index. The resistive index was calculated according to the following formula: Resistive index = 1 – (EDV / PSV). *n* = 24 (aged) and 5 (glaucoma). The data are presented as means ± S.E.M. *P < 0.05; **P < 0.01.

The angle of the anterior chamber was normal in glaucomatous marmosets (Fig. 3a–d) as were the axial lengths of the eyeballs (Fig. 3e), suggesting that the abnormalities in the optic disc of glaucomatous marmosets were not due to angle closure or high myopia^18^. In addition, the iridocorneal angle in glaucomatous marmosets was well formed with an obvious Schlemm’s canal (Fig. 3f). The IOP in glaucomatous marmosets was similar to that in aged controls (Fig. 3g). In addition, the cerebrospinal fluid (CSF) pressure of glaucomatous marmosets was lower compared with controls (Fig. 3h). We next examined the ophthalmic arterial blood flow using color Doppler imaging. Resistive index is a useful measure of vascular damage. In humans, resistive index correlates with aging, visual impairment observed in NTG patients and systemic atherosclerosis^19–21^. Resistive index is calculated by [1 – (EDV/PSV)], where PSV is the peak systolic velocity and EDV is the end diastolic velocity (Fig. 3i). We found that the resistive index in aged controls was significantly higher than that in glaucomatous marmosets (Fig. 3j). These data indicate that arteriosclerosis, an aging hallmark in human, can be monitored in marmoset eyes in a non-invasive method *in vivo*, allowing objective evaluation of changes associated with disease. Taken together, by these criteria, the glaucomatous marmosets present with pathology comparable to human NTG.

**Figure 4:**
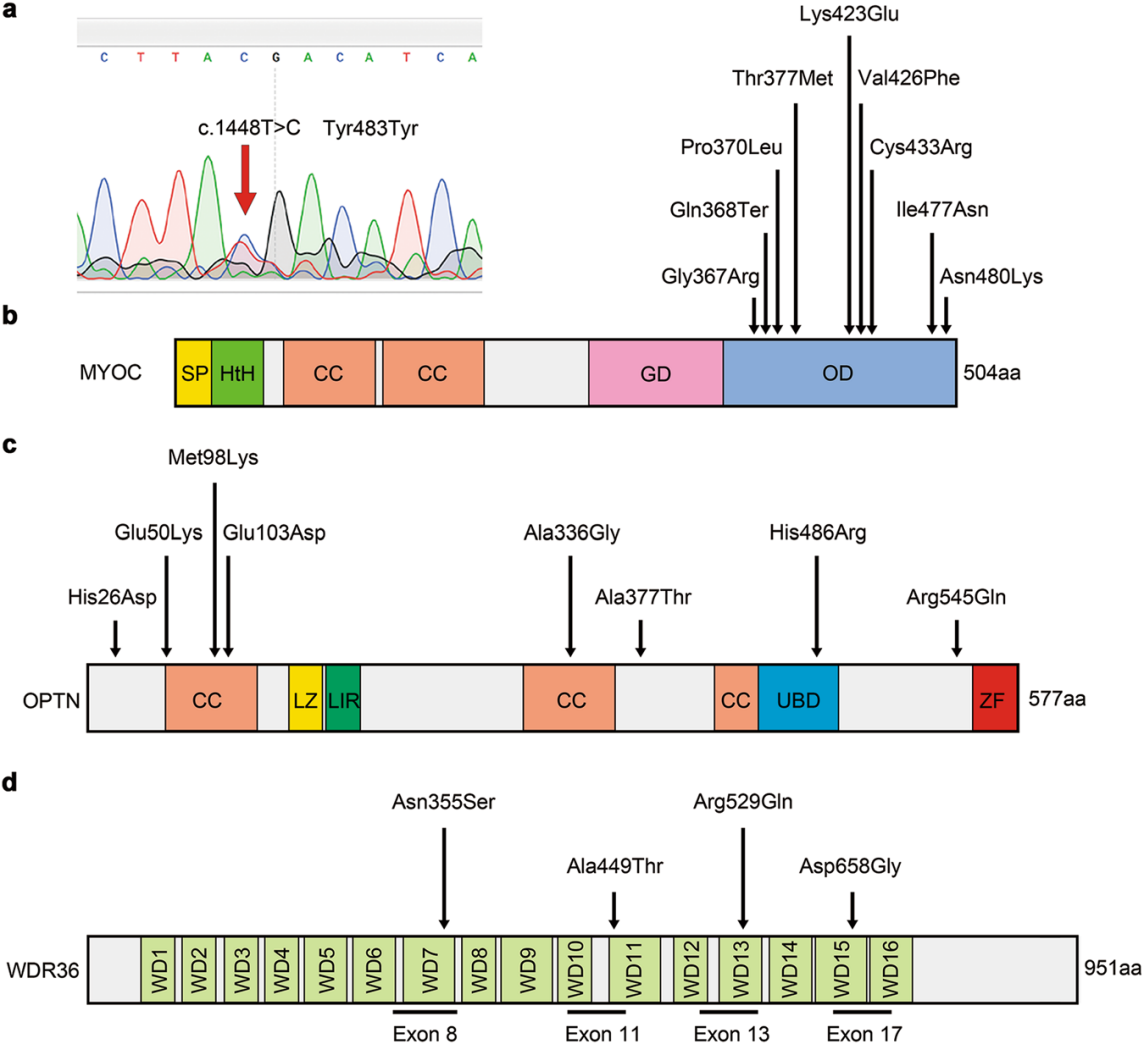
Genomic analysis of glaucomatous marmosets for glaucoma associated genes. **(a)** A single base substitution in the MYOC gene was identified in the glaucomatous marmoset (No. 34 in Supplementary Table 1). However, this is a silent mutation: a single base mutation that does not alter protein production. **(b)** A domain structure of the human MYOC gene with indication of the mutations associated with glaucoma. All the reported human mutation sites of the MYOC gene were analysed in the glaucomatous marmosets (No. 33–36). Gene mutations associated with glaucoma were not detected. SP: signal peptide, HtH: helix-turn-helix, CC: coiled coil, GD: globular domain, OD: olfactmedin domain. **(c,d)** Domain structures of human OPTN **(c)** and WDR36 **(d)** genes and location of identified mutations. All the reported human mutation sites of the OPTN gene and exons 8, 11, 13, and 17 of the WDR36 gene were analysed in the glaucomatous marmosets (No. 33–36). Gene mutations associated with glaucoma were not detected. CC: coiled coil, LZ: leucine zipper, LIR: LC3-interacting region, UBD: ubiquitin-binding domain, ZF: zinc finger, WD: WD repeat domain.

We next examined whether these marmosets possessed any gene mutations associated with glaucoma: *MYOC*, *OPTN* and *WDR36* (encoding the proteins myocilin, optineurin and WD repeat domain 36, respectively)^22–25^. Genetic analyses of glaucomatous marmosets did not find mutations in the aforementioned genes (Fig. 4), indicating that these marmosets present with spontaneous NTG, which is the most common form of human NTG.

### Disease progression of a glaucomatous marmoset during a one-year follow-up

**Figure 5:**
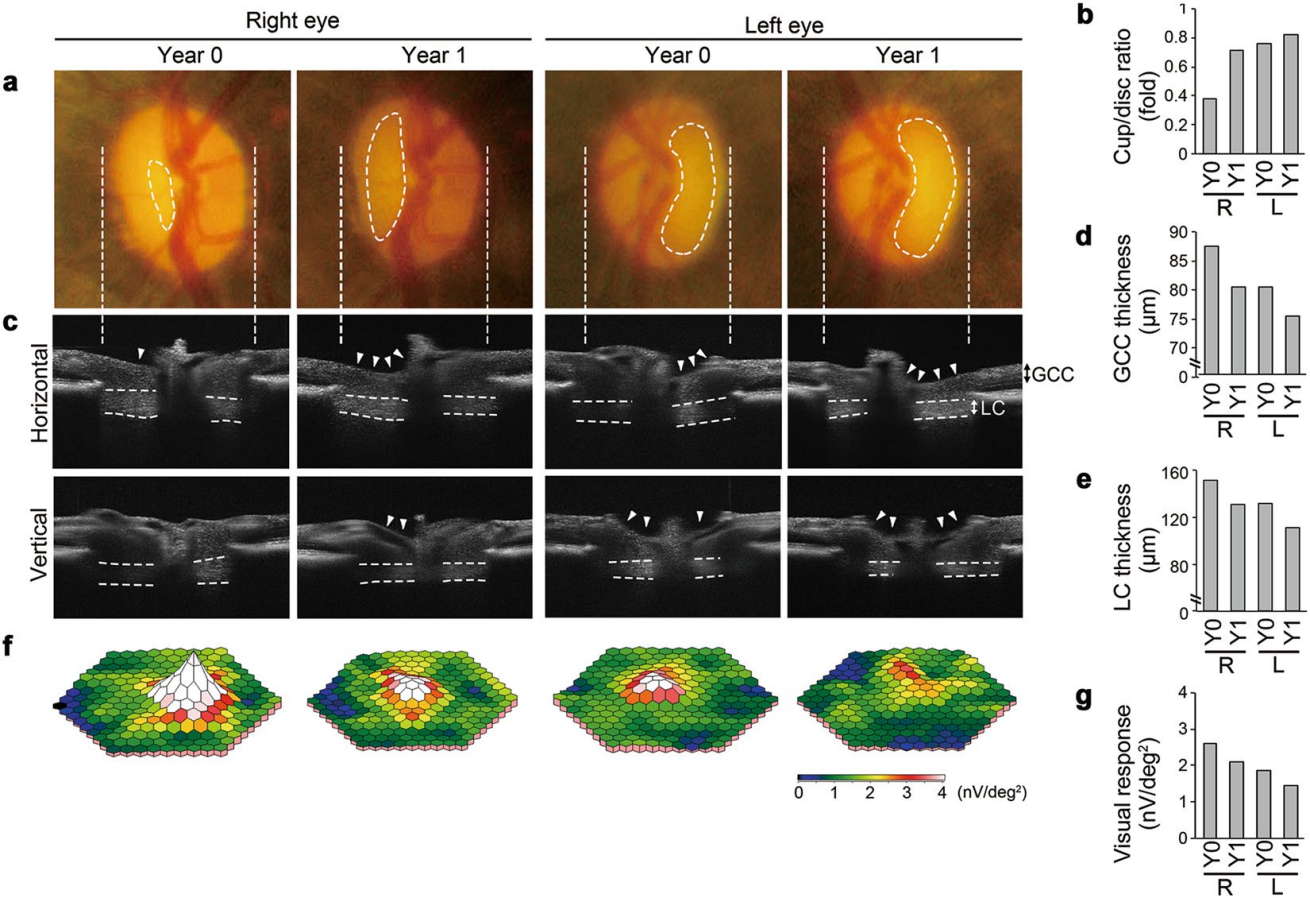
Follow-up studies of a glaucomatous marmoset over 12 months. **(a)** Ocular fundus photographs of initial examination (Year 0) and one year later (Year 1) in the glaucomatous marmoset (No. 34 in Supplementary Table 1). Dotted lines indicate the cupping of the optic disc. **(b)** Quantitative analysis of the vertical cup/disc ratio. R: right eye, L: left eye, Y0: year 0, Y1: year 1. **(c)** *In vivo* imaging of the optic disc by the horizontal and vertical scan through the centre of the optic disc by SD-OCT. Arrowheads indicate the cupping of the optic disc and dotted lines indicate the LC. **(d)** Longitudinal evaluations of the GCC thickness by a circular scan around the optic disc by SD-OCT. **(e)** Quantitative analysis of the LC thickness by cross-sectional images of the optic disc by SD-OCT. **(f)** Three-dimensional plots of the retinal responses as examined by mfERG. Values are given in nV per square degree (nV/deg^2^). **(g)** Quantitative analysis of the visual responses in **(f)**.

When we followed up with one of the glaucomatous marmosets after 12 months, its non-diseased eye had also developed glaucoma-like features (No. 34 in Supplementary Table 1). Fundus imaging at an initial examination (Year 0) detected optic disc cupping and vascular abnormalities (bending and relocation) around the cupping region in the left eye, while the right eye appeared comparable to a normal aged marmoset eye (Fig. 5a). One year later (Year 1), we found that optic disc cupping and relocation of the blood vessels were more prominent in the left eye and interestingly, the right eye also showed glaucoma-like changes (Fig. 5a). *In vivo* imaging with SD-OCT visualized clear optic disc cupping in the left eye at Year 0 and its exacerbation at Year 1, while the right eye developed glaucoma-like characteristics between Year 0 and 1 (Fig. 5b,c). Furthermore, reduction in the thickness of the GCC and LC was observed in the left eye at Year 0, which was further decreased at Year 1, and for the right eye, the thickness of the GCC and LC was comparable to the aged group at Year 0, but it was reduced at Year 1 (Fig. 5c–e). We next determined changes in visual function over one year. Electrophygiological responses from the left eye were lower than average aged marmosets at Year 0 and they further decreased at Year 1, while the responses from the right eye at Year 0 were comparable with average aged marmosets, but they were reduced at Year 1 (Fig. 5f,g). Unfortunately, we were unable to follow up the other 3 glaucomatous marmosets due to their old age and need for other experiments. Taken together, these data indicate that this marmoset presents with binocular glaucoma-like degeneration. Such laterality is also observed in human glaucoma and is a useful trait from an experimental point of view because the vulnerable eye can be used to examine if a novel therapeutic strategy is capable of preventing disease onset or progression.

### Effects of glaucoma-like retinal degeneration on the central visual system in marmosets

The visual pathway relays information from the retina to the lateral geniculate nuclei (LGN), then LGN to the primary visual cortex. Recent studies suggest a possibility that progressive glaucoma induces not only RGC death and optic nerve axon loss, but also neurodegeneration throughout the central visual system such as the LGN and primary visual cortex^26^. Since the brain of a marmoset is more complex than rodents and more similar to humans^27–29^, marmosets are a good model for studying the brain pathology. We therefore explored any changes in the brain of the glaucomatous marmosets.

**Figure 6:**
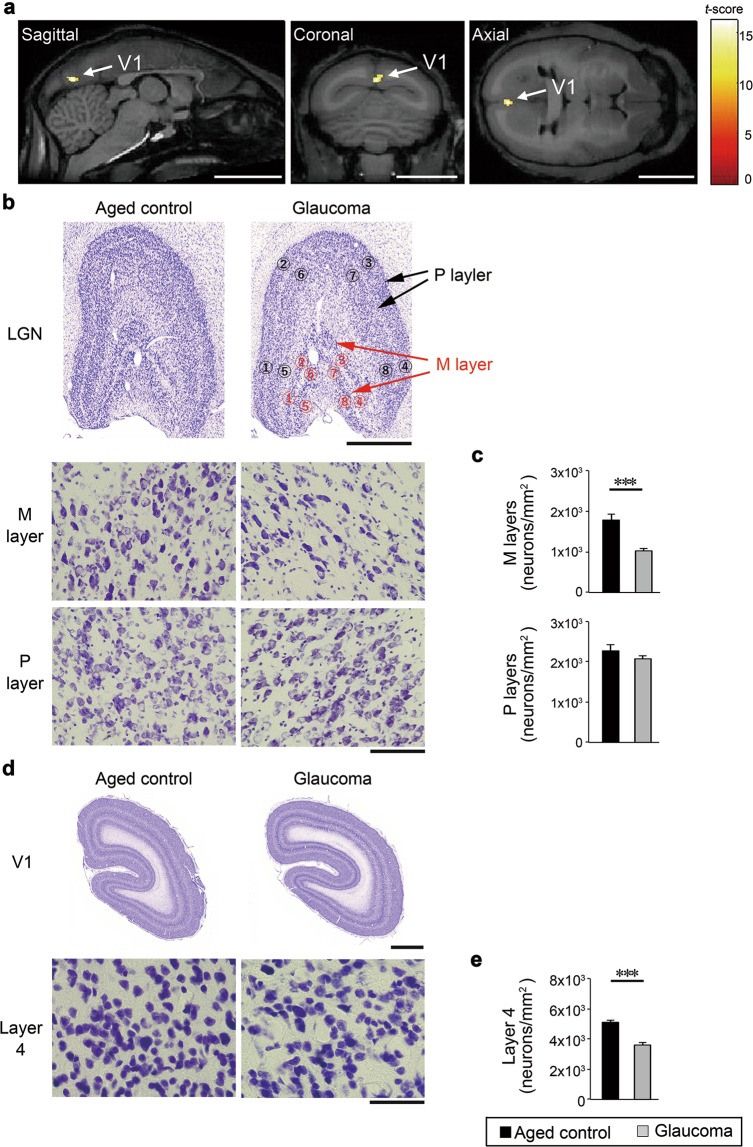
Degeneration of the central visual system in glaucomatous marmosets. **(a)** Volume loss in the primary visual cortex, also known as visual area 1 (V1) in the glaucomatous marmosets comparison with normal aged controls. The color scale bar in voxel-wise statistics indicates the *t*-score and the areas of the gray matter with significant volume reduction were superimposed on the template brain (*P* < 0.001, Student’s *t* test). *n* = 4 (aged) and 3 (glaucoma). Scale bar: 10 mm. **(b)** Representative images of coronal sections of the lateral geniculate nuclei (LGN) (upper) and magnified images of the magnocellular (M)- and parvocellular (P)-layers of the LGN (area No. 7) in aged and glaucomatous marmosets (lower). Scale bar: 1 mm and 100 µm for upper and lower panels, respectively. **(c)** Quantitative analyses of the cell number in the LGN. *n* = 3 per group. **(d)** Representative images of coronal sections of the visual cortex (upper) and magnified images of the layer 4 of the visual cortex in aged and glaucomatous marmosets. Scale bar: 1 mm and 50 µm for upper and lower panels, respectively. **(e)** Quantitative analyses of the cell number in the layer 4 of the visual cortex. *n* = 3 per group. The data are presented as means ± S.E.M. ***P < 0.001.

*In vivo* imaging with magnetic resonance imaging (MRI) and voxel-based morphometry (VBM) from a total of 7 marmosets revealed significant volume loss in the primary visual cortex, also known as the visual area 1 (V1) in the glaucomatous marmosets compared with controls (Fig. 6a). These imaging profiles and those of advanced POAG patients are strikingly similar^30^. Studies suggest that in the LGN, the magnocellular (M) layer is damaged earlier than the parvocellular (P) layer in glaucoma patients^31,32^. When we examined the cell number in the M- and P-layers, we found that the cell number in the M layer in glaucomatous marmosets was significantly decreased compared with controls, while the cell number in the P layer was not altered in glaucomatous marmosets (Fig. 6b,c). We further examined the layer 4 of the primary visual cortex, and found that the cell number in the layer 4 in glaucomatous marmosets was significantly decreased compared with controls (Fig. 6d,e). Our data captured atrophy of the central visual system in glaucomatous marmosets both *in vivo* and histologically.

### Oxidative stress is increased and brain-derived neurotrophic factor (BDNF) and TrkB are decreased in glaucomatous marmosets

**Figure 7:**
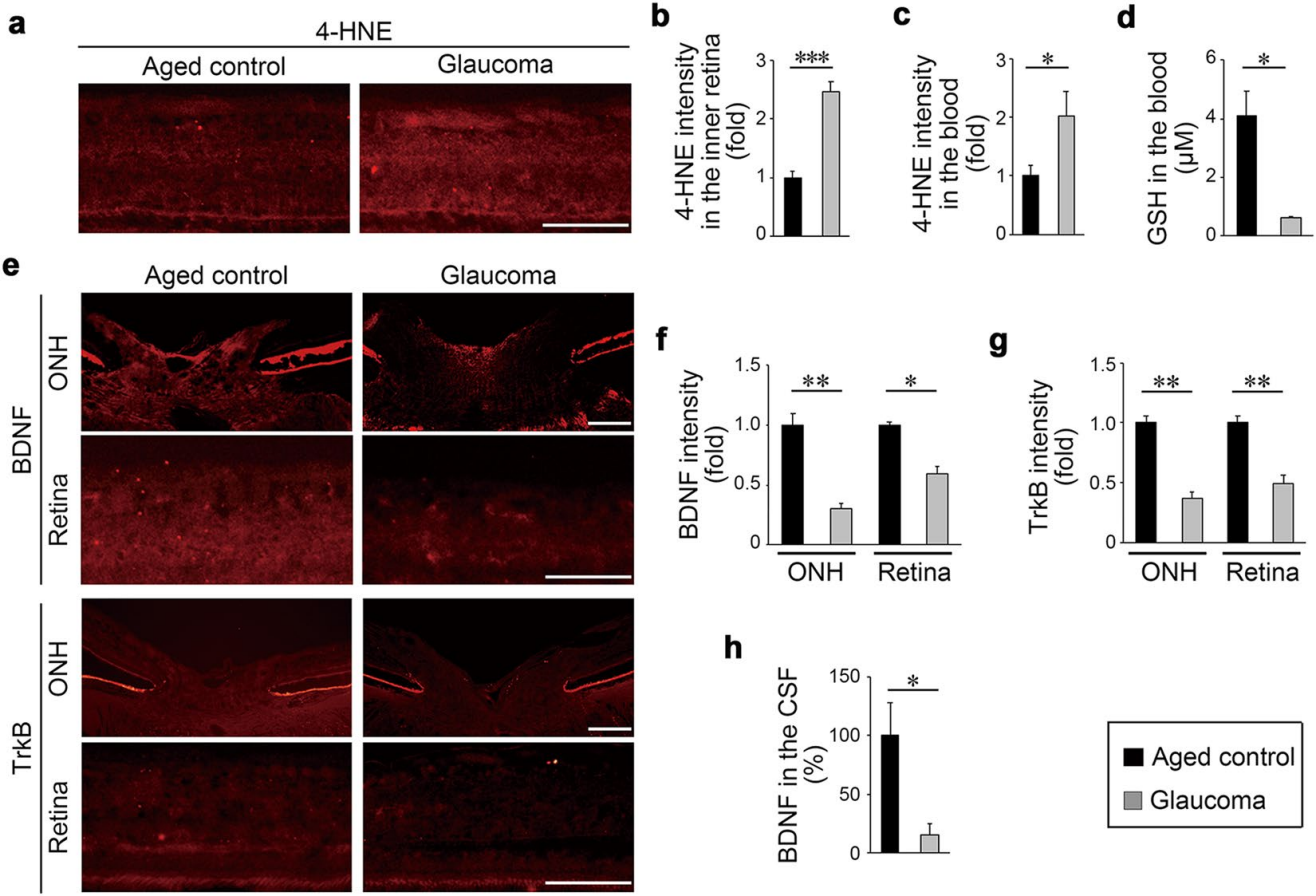
Increased oxidative stress and decreased BDNF/TrkB expression in glaucomatous marmosets. **(a)** 4-HNE expression in the retina detected by immunohistochemistry. Scale bar: 100 µm. **(b)** Quantitative analyses of the intensity of 4-HNE. *n* = 3 per group. **(c)** 4-HNE expression in the blood detected by immunoblot analyses. *n* = 9 (aged) and 3 (glaucoma). **(d)** GSH concentrations in the blood. *n* = 4 (aged) and 3 (glaucoma). **(e)** BDNF and TrkB in the optic nerve head (ONH) and retina detected by immunohistochemistry. Scale bar: 100 µm. **(f,g)** Quantitative analyses of the intensity of BDNF **(f)** and TrkB **(g)**. *n* = 3 per group. **(h)** BDNF concentrations in the CSF. *n* = 3 per group. The data are presented as means ± S.E.M. *P < 0.05; **P < 0.01, ***P < 0.001.

Oxidative stress is one of the pathogenic factors in glaucoma^33,34^. Therefore, we examined the oxidative stress levels of glaucomatous marmosets by measuring expression of 4-hydroxy-2-nonenal (4-HNE). Retinal expression of 4-HNE, particularly at the inner retina, was remarkably high in glaucomatous marmosets (Fig. 7a,b). Similarly, blood 4-HNE expression in glaucomatous marmosets was significantly higher than that in controls (Fig. 7c). We also examined the blood levels of the antioxidant glutathione (GSH), a reduction of which is observed in glaucoma patients^35^. The blood GSH level was significantly decreased in glaucomatous marmosets (Fig. 7d).

The BDNF-TrkB signalling pathway plays a major role in neuroprotection, particularly for RGCs^36,37^. BDNF is reduced with aging in humans^38^ and reduction of BDNF and its receptor TrkB is observed in glaucoma patients^39^. Consistent with these reports, the BDNF and TrkB expression levels in the optic nerve head and retina, particularly in the inner retina, were significantly decreased in glaucomatous marmosets (Fig. 7e–g). We also measured the BDNF level in the CSF and found that it was significantly lower in glaucomatous marmosets than in controls (Fig. 7h). These results indicate that some of the key biochemical changes associated with human glaucoma are observed in glaucomatous marmosets.

## Discussion

Herein, we report that approximately 11% of aged marmosets examined presented with spontaneous NTG; a rate very similar to that found in humans. To our knowledge, this is the first report to map retinal structure and function in the same subject in the common marmoset. The major advantage of marmosets over mice as a model for studying eye diseases is that they possess the LC and the macula, neither of which are present in mice^11,40^. In glaucomatous marmosets, reduction in the LC thickness and atrophy of the primary visual cortex, which are consistent with human glaucoma patients, were detected^41,42^. Furthermore, the short lifespan of marmosets compared with other non-human primates is another advantage. We found that unilateral optic neuropathy developed into severe bilateral glaucoma within one year; such disease progression is also observed in human glaucoma, but over a much longer time period. The aging processes occurring over the course of 5 years in marmosets may be equivalent to the processes occurring over 10 years in macaques and 25 years in humans (these are estimations based on their respective lifespans).

Systemic oxidative stress levels are associated with decreased ocular blood flow in NTG patients^34,40,43^. Our data suggest the presence of decreased blood flow and increased oxidative stress in glaucomatous marmosets. Furthermore, we found that the expression levels of BDNF and TrkB are reduced in the optic nerve head and retina in glaucomatous marmosets, consistent with the data gathered from observation of human glaucoma patients^39^. However, other researchers previously reported accumulation of TrkB at the optic nerve head in an experimental glaucoma model using cynomolgus monkeys (*Macaca fascicularis*)^44^. These discrepancies may have arisen due to the difference between the spontaneous (chronic) and experimental (acute) glaucoma, and/or due to the difference in the age of the experimental animals; they examined juvenile monkeys while we examined aged marmosets. Neurotrophins are a therapeutic candidate for glaucoma and studies show that BDNF eye-drops rescue visual responses in DBA/2J mice, a mouse model with high IOP glaucoma^45,46^. BDNF gene therapy may be useful for patients who show decreased BDNF levels, and recent development in gene therapy that increases both BDNF and TrkB expression appears very promising^47^. Interestingly, gene therapy with ciliary neurotrophic factor (CNTF) was very effective in experimental rat glaucoma^48^ and topical application of nerve growth factor (NGF) improved visual function in glaucoma patients^49^, suggesting trophic factors may be useful for the treatment of glaucoma. In fact, clinical trials are underway for encapsulated cell therapy with CNTF, and for recombinant human NGF eyedrops (ClinicalTrials.gov Identifier: NCT02862938 and NCT02855450). It is possible that non-IOP targeting drugs for treatment of glaucoma will be clinically available very soon.

In this study, we found aged marmosets that develop NTG-like retinal degeneration naturally, and we demonstrate that the pathological features are similar in marmoset and human glaucoma. Unfortunately, the incidence of these naturally occurring NTG marmosets may be too low to be an effective animal model for testing therapeutic interventions. Excitingly, generation of the transgenic marmoset was recently reported^50,51^ and this technology will be a powerful tool for medical research of various diseases. We have previously reported that the loss of glutamate transporters in mice leads to phenotypes similar to NTG^9^, and we are now planning to target these genes and generate marmoset models of NTG. Genetic manipulation of the common marmoset raises high hopes for great advances in medicine and will no doubt provide a beneficial outcome for public health.

## Methods

### Animals

Experiments were performed using 36 (15 male and 21 female) adult common marmosets (*Callithrix jacchus*) (Supplementary Table 1). Marmosets were derived from a breeding colony at the Tokyo Metropolitan Institute of Medical Science and Central Institute for Experimental Animals. Animal experiments were approved by the Institutional Animal Care and Use Committee of the Tokyo Metropolitan Institute of Medical Science (Approval number TMiMS: 16082) and the Central Institute for Experimental Animals (Approval number CIEA: 16055A). All animals were handled with care and all animal experiments were performed in accordance with the ARVO Statement for the Use of Animals in Ophthalmic and Vision Research and the guidelines for the care and use of animals at both institutes. Experiments were performed under anaesthesia as previously reported^52,53^. The IOP measurements were made using an applanation tonometer (Tono-Pen XL, Medtronic Solan, Jacksonville, FL, USA), as previously reported^54,55^. We could not measure IOP in one eye (No. 12; an aged marmoset) due to corneal opacity.

### Ophthalmoscopy, SD-OCT and mfERG

Pupils were dilated with 0.5% phenylephrine hydrochloride and 0.5% tropicamide. Ophthalmoscopy for screening marmoset eyes was performed with an ophthalmoscope (OMEGA 500, HEINE Optotechnik, Herrsching, Germany). Ocular fundus photographs were obtained using a small animal fundus camera (Genesis-D, Kowa, Tokyo, Japan). SD-OCT (RS-3000, Nidek, Aichi, Japan) examinations were performed as previously described^55,56^ with some modifications. A 40-D adaptor lens was placed on the objective lens of the multiline OCT to focus on the marmoset retina. For imaging of the retinal layers, line scans and circular scans around the optic disc were performed, and the thickness of the GCC was measured. In the disc map 3D scans, optic nerve head analysis was conducted to calculate the vertical cup to disc ratio^57,58^.

In the disc map 3D scans, optic nerve head analysis was conducted using both automatic and manual definitions of the disc margin with the instrument’s software. The disc circumference was determined by tracing the inner edge of retinal pigment epithelium. Then, parallel to this circle and 175 µm towards the vitreous body, the cup circumference was determined at the point that intersect with the inner limiting membranes^58^.

The thickness of the LC was measured by cross-sectional images of the optic disc and calculated with the instrument’s software as previously reported^59^. The images of the anterior chamber were taken in a bright room by using the same SD-OCT with an anterior segment adaptor. Measurement of the anterior chamber angle (ACA) and angle opening distance (AOD) were calculated with the instrument’s software as previously reported^60,61^. AOD was determined by the length of a line drawn from the anterior iris to the corneal endothelium, perpendicular to a line drawn along the trabecular meshwork at a given distance from the scleral spur. AOD750 was calculated at 750 µm from the scleral spur and ACA was calculated as the angle of the previous two lines (Fig. 3a).

mfERGs were recorded using a VERIS 6.0 system (Electro-Diagnostic Imaging, Redwood City, CA, USA) as previously reported^9,55,56^ with modifications. A contact electrode (Mayo, Nagoya, Japan) was used and the visual stimulus consisted of 61 hexagonal areas scaled with eccentricity.

### Measurement of the axial length

After the placement of an eyelid speculum to a marmoset, a sterile ophthalmic gel was applied to the cornea, and the B-mode probe was placed on the gel to obtain the horizontal axial scan passing through the optic nerve and macula using a color Doppler imaging scanner (Aplio 300, Toshiba, Tokyo, Japan). The length between the corneal cap and macula was estimated with the instrument’s software.

### Collection and analysis of CSF

A twenty-seven-gauge butterfly needle connected to a manometer was inserted in the cisterna cerebellomedularis of a marmoset, a manometer was held up and then the CSF pressure was measured by recording the height meniscus of CSF in the manometer tube^62^. CSF collection was performed as previously reported^63^. During the collection, the marmoset was placed in a prone position, and the back of the head and the neck were kept at level. The BDNF concentration in the CSF was measured using an assay kit DBD00 (R&D, Minneapolis, MN, USA) according to the manufacturer’s protocol.

### Doppler imaging of the ophthalmic artery

The flow velocity of the ophthalmic artery of a marmoset was determined using a color Doppler imaging scanner (Aplio 300, Toshiba). Throughout the entire procedure, the systolic blood pressure (117.8 ± 7.5 mmHg), diastolic blood pressure (65.4 ± 6.2 mmHg) and heart rate (164.6 ± 9.4 beats/min) were measured automatically^64^.

### Genotyping from marmoset fingernails

DNA from marmoset blood cannot be used for genetic analyses as hematopoietic cells are exchanged between siblings *in utero*, resulting in chimerism^65,66^. Therefore, we extracted DNA from marmoset fingernails^67^. We collected fingernails without the hyponychium. Approximately 1.5- or 2-mm fingernail fragments were clipped from the finger claw. The crude DNA solution was extracted from each sample using the alkaline lysis method. A piece of fingernail was immersed in a 100 µl aliquot of 50 mM NaOH and boiled at 95 °C for 10 min. Then, 10 µ1 of 1 M Tris-HCl (pH 8.0) was added and mixed. This crude DNA extracted solution was used as the DNA source. PCR was performed with the KOD FX polymerase (Toyobo, Osaka, Japan), using the sense and antisense primers described in Supplementary Table 2. PCR products were sequenced using a Big Dye V3.1 Terminator Kit (Applied Biosystems, Foster City, CA, USA) and an ABI Prism 3100 DNA sequencer (Applied Biosystems). Major genetic abnormalities in human glaucoma (*MYOC*, *OPTN* and *WDR36*) were examined in glaucoma marmosets with reference to the GenBank as standard DNA data (GenBank accession number; XM_008985025, XM_002750043 and XM_008991713, respectively). Based on previous reports, we searched for the following mutations (Fig. 4): *MYOC*- Gly367Arg^68^, Gln368Ter, Pro370Leu^69^, Thr377Met^70^, Lys423Glu^71^, Val426Phe^72^, Cys433Arg^73^, Ile477Asn^74^ and Asn480Lys^75^; *OPTN*- His26Asp^76^, Glu50Lys, Met98Lys, Arg545Gln^24^, Glu103Asp, His486Arg^77^, Ala336Gly and Ala377Thr^78^; *WDR36*- Asn355Ser, Ala449Thr, Arg529Gln and Asp658Gly^79^.

### *In vivo* imaging of the brain

MRI study and VBM analysis of the brain were carried out in 7 marmosets (Supplementary Table 1) as previously reported^80^. All MRIs were performed using a 7-T Biospec 70/16 MRI system (Bruker, Billerica, MA, USA). VBM analysis was performed with SPM12 (Wellcome Trust Centre for Neuroimaging, UCL Institute of Neurology). Modulated normalization images of the grey matter were obtained with diffeomorphic anatomical registration through exponentiated lie algebra (DARTEL), multiplied by the Jacobian determinants derived from the spatial normalization, and were used to evaluate the local tissue volume of the grey matter. Finally, the group difference between the glaucomatous marmosets and aged controls of gray matter volume were examined voxel-wise using the two-sample *t*-test model in the SPM12 software with sex and age of subjects added to the model as covariates. Areas of the brain that demonstrated significant differences between groups were visualized by the SPM12 software with a pseudo-color.

### Histological and morphometric studies

Paraffin sections (7 µm-thick) of eyeball specimens were cut through the optic nerve and of optic nerves were cut cross-sectionally, then stained with hematoxylin and eosin (H&E). The cell number in the GCL was counted from one ora serrata through the optic nerve to the other ora serrata. as previously described^81,82^. Five sections per eye were counted and the average values are presented. The GCC and LC thickness were analysed with the microscope software (Keyence BZ-2 system, Keyence, Osaka, Japan). Brain specimens were serially sliced into coronal sections (30 µm) on a freezing microtome and stained with cresyl violet for Nissl staining^83^. To estimate the level of degeneration in the central visual system, the cell density in the M- and P-layers in the LGN and the layer 4 in the visual cortex (Brodmann area 4C) were quantified by counting all cells located within the area 370 × 270 × 30 µm^29,84^. Total cell counts were derived from the average of four regions per layer in the LGN^28,29^ and three regions in layer 4 of the visual cortex in each of the three adjacent sample sections, and performed automatically with the microscope software (Keyence).

### Immunohistochemistry

Frozen sections (10 µm) were incubated with a primary antibody against 4-HNE (1:200; MHN-100P, Japan Institute for the Control of Aging, Shizuoka, Japan), BDNF (1:200; sc-546, Santa Cruz, Santa Cruz, CA, USA) or TrkB (1:200; sc-8316, Santa Cruz). Immunofluorescence was imaged using a BX51 microscope (Olympus, Tokyo, Japan) and the intensities of 4-HNE, BDNF and TrkB at the optic nerve head and inner retina were analysed using a NIH ImageJ software 1.46r (http://imagej.nih.gov/ij/; provided in the public domain by the National Institutes of Health, Bethesda, MD, USA)^85^. Three sections per eye were analysed and the fluorescent images in each sample were collected under the same relative gain and threshold settings.

### GSH and 4-HNE assays

The GSH concentration in the blood was measured using an assay kit STA-312 (Cell Biolabs, San Diego, CA, USA) according to the manufacturer’s protocol. The 4-HNE expression in the blood was examined by immunoblot analysis with an antibody against 4-HNE (1:1000). The intensity of 4-HNE was analysed using a NIH ImageJ software 1.46r.

### Statistics

Data are presented as means ± SEM. All data collection was performed blind to sample identity. When statistical analyses were performed, the Student’s *t*-test was used. *P* < 0.05 was regarded as statistically significant. JMP version 13.1.0 (SAS Institute Inc., Cary, NC, USA) was used for the statistical analyses. We indicated one eye as n = 1 and so the n does not reflect on the number of animals. This is because glaucoma-like characteristics were often observed in one eye and not the other of the same animal. This phenomenon that glaucoma-like degeneration does not occur in both eyes at the same time is also commonly seen in humans.

## Supplementary information

Supplementary Information

## Data Availability

The datasets generated during and/or analysed during the current study are available from the corresponding author on reasonable request.
