# Supplementary material for: Normal tension glaucoma-like degeneration of the visual system in aged marmosets
Source: Sci Rep. 2019 Oct 16;9:14852. doi: 10.1038/s41598-019-51281-y (PMC6795850; doi:10.1038/s41598-019-51281-y)

Supplementary Information for

Normal tension glaucoma-like degeneration of the visual system in aged marmosets

Takahiko Noro[1,2,3], Kazuhiko Namekata[1], Atsuko Kimura[1], Yuriko Azuchi[1], Nanako Hashimoto[4], Keiko Moriya-Ito[4,5], Yuji Komaki[2], Chia-Ying Lee[2], Norio Okahara[2], Xiaoli Guo[1], Chikako Harada[1], Euido Kim[1,3], Tadashi Nakano[3], Hiroshi Tsuneoka[3], Takashi Inoue[2], Erika Sasaki[2], Hironobu Tokuno[5] and Takayuki Harada[1]

[1]Visual Research Project, Tokyo Metropolitan Institute of Medical Science, Tokyo, Japan.

[2]Central Institute for Experimental Animals, Kawasaki, Japan.

[3]Department of Ophthalmology, The Jikei University School of Medicine, Tokyo, Japan.

[4]Center for Basic Technology Research, Tokyo Metropolitan Institute of Medical Science, Tokyo, Japan.

[5]Laboratory of Brain Structure, Tokyo Metropolitan Institute of Medical Science, Tokyo, Japan.

*Corresponding author: **Takayuki Harada, M.D. Ph.D.**

Visual Research Project, Tokyo Metropolitan Institute of Medical Science

2-1-6 Kamikitazawa, Setagaya-ku, Tokyo 156-8506, Japan.

E-mail: harada-tk@igakuken.or.jp

Supplementary Table 1. List of marmosets examined in this study

	ID	Sex	Age (years)	Body Weight (g)	IOP		Axial Length		Ophthal-moscopy	OCT	mfERG	Doppler Imaging	Blood Test	CSF	MRI
					R	L	R	L							
	1	F	8	310	17	22	8.9	9	○	○	○	○	○		
	2	F	8	358	22	23			○					○	
	3	F	9	368	23	25	9.6	8.8	○	○	○	○	○		
	4	M	9	362	20	19			○						
	5	M	10	368	17	18	9.5	9.7	○	○	○				
	6	F	10	369	15	14	9.1	9.5	○	○	○				
	7	F	10	354	21	21			○					○	
	8	M	10	377	22	22			○						
	9	F	10	348	19	17	9.2	9.8	○	○	○	○	○	○	
	10	M	10	354	21	20	9.8	10	○	○	○	○			○
	11	F	10	359	20	19			○						
	12	F	10	331	26	—			○						
	13	F	10	442	25	30			○						
	14	M	10	345	23	26			○						
	15	F	10	399	19	23			○						
Aged	16	F	10	389	17	16			○						
n = 32	17	M	11	301	16	20	10.2	10.3	○	○	○	○	○	○	
	18	M	11	343	21	20			○						
	19	M	11	326	20	25	10.2	10	○	○	○	○	○	○	○
	20	F	11	367	20	19	9.6	9.7	○	○	○	○	○	○	○
	21	F	11	369	22	21	10	9.7	○			○	○		
	22	F	11	334	27	30			○						
	23	M	12	333	22	25	10.1	9.9	○	○	○				
	24	F	12	329	30	29			○						
	25	F	13	358	24	22	10	10.4	○	○	○	○	○		○
	26	M	13	308	20	20	10.1	10.1	○	○	○	○	○		
	27	M	13	334	22	19			○						
	28	F	14	480	24	31			○						
	29	M	14	360	17	25			○						
	30	M	15	332	19	17	10.1	10.6	○	○	○	○			
	31	M	15	272	23	20			○						
	32	F	16	372	24	23	10.2	10.7	○	○	○	○			
Average			11.2	355	21.2	22.0	9.8	9.9							
SE			0.4	6.8	0.6	0.8	0.1	0.1							
	33	M	12	347	21	23	10.3	10.4	○	○	○	○	○	○	○
Glaucoma	34	F	12	434	17	17	9	9.1	○	○	○	○	○	○	
n = 4	35	F	13	298	22	24	10.1	10.2	○	○	○	○	○	○	○
	36	F	13	366	18	19			○	○	○				○
Average			12.5	361	19.4	20.7	9.8	9.9							
SE			0.3	28.2	1.2	1.6	0.4	0.4							

Supplementary Table 2. Primer sequences used for PCR

Gene	Exon	Sequences
MYOC	1	F: TCTTGCTGGCAATGTGAAGGCAACC<br>R: TGCTATGAGCCAGATCACCTGCTGA
GenBank accession number XM_008985025	2	F: TTCAAGCGATTCTCCTGCCTCCA<br>R: ATTCTTCATGACCAGTAAGTGGG
	3	F: AATGGTCACAGTCTGTGTTT<br>R: GTCAGATGGTGACCATGCACATCCT
OPTN	2	F: GATCATCATGAGGTCATACCCTTGT<br>R: ATGGCATTACCCGAAGGCCTGGGAT
GenBank accession number XM_002750043	3	F: AATGAAGTCCACTCTTCCTGGTGTG<br>R: GGCTCCTTAGCAAACGCATCCAGTG
	4	F: CCCAGAGCTCTGGATTACAGGTGTA<br>R: TGAGCCGAGATCGCATCATTGCACT
	5	F: GAAGGTTCCTTGCGTTACATGTCAC<br>R: GCAGGCCATTACACTTGAGTTCCTG
	6	F: CACCAGTGGTGTATGAGCATTCCAC<br>R: ACCGCTGCTCGGGAAGACAATTTGG
	7	F: ACAGTTGGCTTAAAGGTAGGCCTTC<br>R: GAATTCGGTGGCTGGACTACTCTCA
	8	F: CTGTGATCTCTGAACCTGGAGTAAG<br>R: CAGCTCTGTAGAGCTCTCACTTAGG
	9	F: TGCATCACTGCATGAGAAGCGCCTT<br>R: ATCTGAGAAGTCTCCTAGGACTCCT
	10	F: CCACTTGAGTGTTCAGAAGGTTGGG<br>R: TCCACCTCTTCCTGAAGTCAGTGGA
	11	F: GTGCTGAGATTCCAGGCATAAGCCA<br>R: GCCTTGCTCAGCTATGGTTCTGGAA
	12	F: GTAGAGAATCCACAGTCTTCTAGGT<br>R: CTACAGCTAATGCTGATGTGAGCTC
	13	F: CAGGGTTGTAGAACGTCATATGTAG<br>R: TTGCACTCCAGCCTGGGTAACAAGA
	14	F: TGCAGTGAGCCAAGATCGCACCACT<br>R: AGTATCTGGGATAACAGGCACGTGC
WDR36	8	F: ATGCACACTCTACAGCAATTGCCGG<br>R: AGCCACTGATCCTGGAATTACAGGC
GenBank accession number XM_008991713	11	F: GACTTCAGAATACCATGTCAGTGAG<br>R: AGATCAGCATGTACCTGGAGTTACT
	13	F: GCCTCTCATCAGGAACTGTAGATGT<br>R: CATGGTCCTTATGATACAACTGCAGT

Supplementary Movies (Please use Adobe Reader to display the movies)

Movie 1.

A representative movie showing the optic disc cupping of an aged marmoset (aged 110 months; No. 3 in Table 1) captured by disc map 3D scans with SD-OCT.

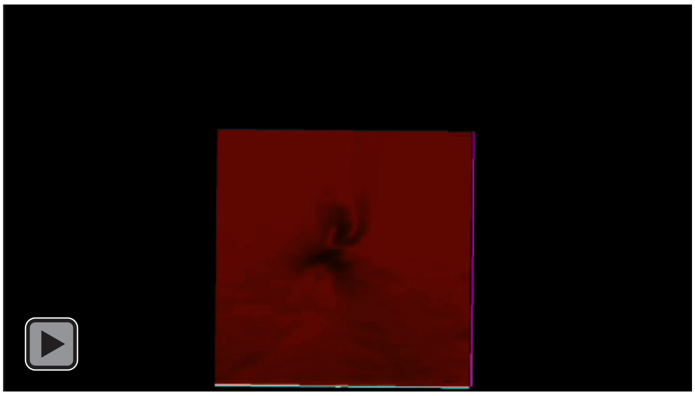

Movie 2.

A representative movie showing the optic disc cupping of the glaucomatous marmoset (aged 157 months; No. 34 in Table 1, Left eye of Year 1 in Figure 5) captured by disc map 3D scans with SD-OCT.

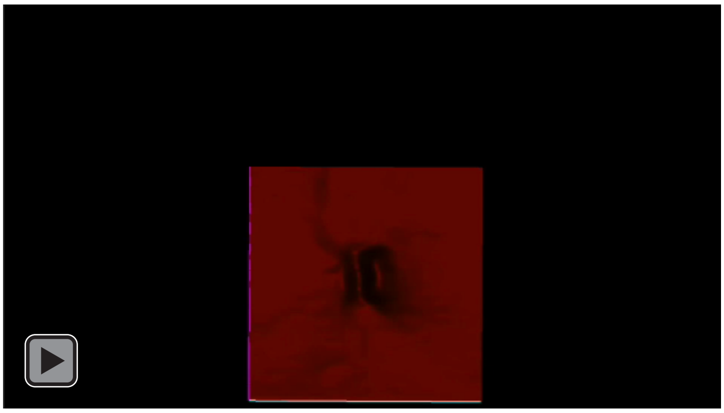

Supplement: Supplementary file 1 — Supplementary Information [file 41598_2019_51281_MOESM1_ESM.pdf]
